# Potential for the embryonic morphogen Nodal as a prognostic and predictive biomarker in breast cancer

**DOI:** 10.1186/bcr3185

**Published:** 2012-05-11

**Authors:** Luigi Strizzi, Katharine M Hardy, Naira V Margaryan, David W Hillman, Elisabeth A Seftor, Beiyun Chen, Xochiquetzal J Geiger, E Aubrey Thompson, Wilma L Lingle, Cathy A Andorfer, Edith A Perez, Mary JC Hendrix

**Affiliations:** 1 Children's Memorial Research Center, Robert H Lurie Comprehensive Cancer Center, Feinberg School of Medicine, Northwestern University, 2300 Children's Plaza, Chicago, IL 60614, USA; 2 Mayo Clinic, 200 First Street SW, Rochester, MN 55905, USA; 3 Mayo Clinic, 4500 San Pablo Road, Jacksonville, FL 32224, USA

## Abstract

**Introduction:**

The re-emergence of the tumour growth factor-beta (TGF-beta)-related embryonic morphogen Nodal has recently been reported in several different human cancers. In this study, we examined the expression of Nodal in a series of benign and malignant human breast tissues to determine the clinical significance of this expression and whether Nodal could represent a potential therapeutic target in breast cancer.

**Methods:**

Tissue sections from 431 therapeutically naive patients diagnosed with benign or malignant breast disease were stained for Nodal by immunohistochemistry and analysed in a blinded manner. The degree of Nodal staining was subsequently correlated with available clinical data, such as diagnoses and disease stage. These tissue findings were further explored in breast cancer cell lines MDA-MB-231 and MDA-MB-468 treated with a Nodal blocking antibody to determine biological effects for target validation.

**Results:**

A variable degree of Nodal staining was detected in all samples. The intensity of Nodal staining was significantly greater in undifferentiated, advanced stage, invasive breast cancer compared with benign breast disease or early stage breast cancer. Treatment of human breast cancer cells *in vitro *with Nodal blocking antibody significantly reduced proliferation and colony-forming ability in soft agar, concomitant with increased apoptosis.

**Conclusions:**

These data suggest a potential role for Nodal as a biomarker for disease progression and a promising target for anti-Nodal therapy in breast cancer.

## Introduction

Various classification schemes have been developed to categorize the heterogeneity of breast cancer in an attempt to better predict disease stage, progression potential and outcome. Traditionally, the diagnosis of breast cancer has been based on histological criteria [1]. Moreover, defined architectural features like those described in the Nottingham Grading system for invasive breast cancer, which includes tubule formation, mitoses and nuclear pleomorphism, are used to classify the differentiation status of breast cancer - with poor differentiation being the hallmark of high grade, more aggressive disease [2]. Over the years, with advances in molecular medicine, the incorporation of markers, such as oestrogen receptor (ER), progesterone receptor (PR) and human epidermal growth factor receptor 2 (HER2), have proven to be especially valuable not only for stratifying certain types of breast cancers in distinct functional groups [3], but also for planning and predicting the outcome with respect to specific treatment options [4,5]. Due to the heterogeneity within specific subgroups of breast cancer and the interobserver variability with detection frequencies, not all breast cancers can be successfully classified into specific risk groups based on the expression profile of these traditional markers alone [3]. For instance, adenoid cystic carcinoma and secretory carcinoma are generally hormone receptor negative, but have favourable prognosis and low recurrence rates [6,7]. Further confounding, the expression profile of these markers not only varies within areas of the same lesion but also during the course of disease in the same patient [8]. Additional studies are needed, therefore, to identify novel biomarkers, based on the molecular underpinnings of disease progression that can be used to predict outcome and response to therapy in a larger population of patients, especially those in the high risk category.

Data from gene expression microarrays have led to the molecular stratification of breast cancer into subgroups, such as luminal and non-luminal tumours [9]. Even with this approach, it is difficult to obtain unequivocal consensus on breast cancer classification among observers [10]. Given the heterogeneous subpopulations comprising breast cancer tissue, a major concern is whether results from this type of broad gene expression analysis can be confidently designated as the 'genetic signature' of a specific breast cancer type and whether this approach can be applied to all breast cancer patients. With the introduction of the cancer stem cell theory, different markers have been reported to identify cancer stem cells (CSCs) with the prospect of exploiting these putative CSCs markers as therapeutic targets [11]. In breast cancer, the role of the 'CD44high/CD24low' expression profile, proposed by some to represent a unique subpopulation of breast CSCs [12], has been challenged by others who postulate that not every breast cancer cell with this particular expression profile possesses the properties of CSCs [13]. This may be due to the genetic heterogeneity within the 'CD44high/CD24low' population [14], which suggests a much broader functional variability for this population. Nevertheless, advances in the field of CSC research have enabled us to characterize the re-emergence of specific embryonic signalling pathways in cancer cells, thus contributing to our understanding of the molecular mechanisms that regulate cancer cell plasticity and aggressiveness [15].

One of the embryonic pathways recently described by our group to have profound implications in cancer progression is Nodal [16,17]. Nodal, a member of the TGF-beta superfamily, plays a major role in the maintenance of pluripotency in embryonic stem cells and subsequent organ development [18-20]. Typically, Nodal binds to the Cripto-1-Alk4/7-ActRIIB receptor complex resulting in Smad2/3/4-dependent gene activation [18]. The Nodal co-receptor, Cripto-1, has been shown to enhance the proliferation, migration and invasion of human breast cancer cells and non-transformed mouse mammary epithelial cells *in vitro *and *in vivo *[21]. In human breast cancers, Cripto-1 expression was also found to correlate with progressive disease [22]. There is also a growing body of evidence which indicates that Nodal expression re-emerges in a number of human cancers, such as melanoma, glioma, breast, endometrial and prostate cancers [16,17,23-25]. Interestingly, in some of these reports, Nodal expression was detected in the context of very low or barely detectable Cripto-1, raising the question as to whether Cripto-1 and Nodal can exert their cancer promoting effects independently of each other [17,26]. Nevertheless, the clinical significance of Nodal expression in cancer has yet to be thoroughly explored. In this study, we examined the expression of Nodal in tissue samples from patients with benign and malignant breast disease and compared the levels of Nodal with the available patient data to determine clinical correlations. These immunohistochemical findings were further explored in human breast cancer cell lines treated with a Nodal blocking antibody to determine biological effects for target validation. Collectively, these data suggest the potential for Nodal as a biomarker for invasive disease and a novel therapeutic target in breast cancer.

## Materials and methods

### Patient samples

Archival formalin-fixed and paraffin-embedded breast tissue sections from 431 patients diagnosed with benign breast disease or breast cancer were obtained from the Mayo Clinic. Patients with a prior diagnosis of any cancer except for basal/squamous skin cancer or concurrent cancer or with a prior history of chemotherapy or radiation therapy were excluded from this study. Slides were labelled with numerical codes and accessed only at the end of the study for statistical analyses with corresponding clinical data. All samples were deprived of any patient identifiers in compliance with the institutional IRB approved study protocol.

### Immunohistochemistry

Four micron thick, formalin-fixed, paraffin-embedded tissue sections were prepared and immunohistochemistry was carried out on a Microm HMS 710i autostainer (Thermo Scientific Lab Vision, Waltham, MA, USA) as previously described [17]. Briefly, following antigen retrieval and blocking steps, sections were incubated in mouse anti-human Nodal antibody (Abcam #55676, Cambridge, MA, USA) at 5 μg/ml for 60 minutes, followed by biotinylated anti-mouse secondary antibody (Biocare Medical, LLC, Concord, CA, USA), and then streptavidin-horseradish peroxidase (Thermo Scientific Lab Vision). Colour was developed with 3,3'-diaminobenzidine substrate (Thermo Scientific Lab Vision) and sections were counterstained with hematoxylin (Biocare Medical, LLC). As a negative control, adjacent serial sections were incubated with ChromPure mouse IgG (Jackson Immunoresearch Labs, West Grove, PA, USA) at the same concentration. Nodal staining was scored as previously described [27] on a scale of 0 to 3 at 10 × and 63 × magnification to determine, respectively, percentage and intensity of Nodal staining within the area of interest (0, no staining; 1 = < 10% or weak; 2 = 10 to 50% or moderate; 3 = > 50% or strong). The two scores were then multiplied to obtain a Nodal Scoring Index (SI). Scoring was performed blinded with respect to clinical information.

### Statistical analyses and clinical correlations

The disease characteristics from each patient's biopsy were classified into different groups: benign versus malignant and benign versus atypia/hyperplasia or versus invasive disease. We assessed the association of patient characteristics of all 431 patients and the pathological characteristics of tumours available from a subgroup of 138 surgical patients. Chi-Square and trend tests across the various groups were used to assess the correlation of Nodal expression with patient's demographic and pathologic characteristics.

### Cell culture and antibody treatment

Human breast cancer cell lines MDA-MB-231 and MDA-MB-468 were obtained from ATCC and cultured in RPMI containing 10% foetal calf serum as previously described [17]. The cell lines were genotyped by short tandem repeat (STR) PCR amplification at the Molecular Diagnostic/HLA Typing Core at Children's Memorial Hospital and authentication confirmed by comparison with ATCC profiles. MDA-MB-231 or MDA-MB-468 cells were treated with a function-blocking rabbit anti-Nodal antibody (Santa Cruz Biotechnology, Santa Cruz, CA, USA; H-110) at 2 μg/ml or 4 μg/ml or with rabbit whole molecule IgG (Jackson Immunoresearch Labs) at 4 μg/ml. For most experiments, antibody was diluted in complete RPMI and added to cells daily for a period of 72 or 96 hours.

### Immunofluorescence

For immunofluorescence experiments, MDA-MB-231 and MDA-MB-468 cells grown on glass coverslips were fixed in ice-cold methanol, blocked with 5% bovine serum albumin in PBS and incubated in rabbit anti-Nodal primary antibody (Santa Cruz Biotechnology; H-110) overnight at 10 μg/ml. Cells were washed with PBS and incubated in donkey anti-rabbit Alexafluor-488 secondary antibody (Invitrogen/Life Technologies, Grand Island, NY, USA) at 4 μg/ml. Coverslips were mounted on glass slides using VectorShield mounting medium containing DAPI (Vector Labs, Burlingame, CA, USA). Staining was visualized on a Zeiss Meta 700 confocal microscope with a 25X Zeiss LD Lci Planapo 25x/0.8 Imm Corr objective and images were captured using Zeiss ZEN software (Carl Zeiss, Inc., Thornwood, NY, USA). Nodal-positive cells were counted in eight random fields of view and the subpopulation calculated as a percentage of DAPI-positive nuclei (+/- SD).

### Western blot analysis

Protein lysates of the cell lines were collected, quantified and subjected to SDS-PAGE gel electrophoresis and Western blotting using standard protocols [17]. Serum-free medium preconditioned for 24 hours was collected from cells cultured in T75 flasks. Medium was concentrated 100-fold using an Amicon Ultra centrifugal filter unit with a 3KD cut-off (EMD Millipore, Billerica, MA, USA). Protein concentrations were quantified and samples examined using standard SDS-PAGE gel electrophoresis and Western blotting methods [17]. All antibodies and working dilutions were as previously described [17,28]. Antigen-antibody complexes were removed from membranes between probing with Western blot stripping buffer (Pierce Thermo Fisher Scientific, Rockford, IL, USA). Protein expression relative to loading control was calculated from an average of three independent experiments using densitometric analysis (NIH ImageJ for Windows software; National Institutes of Health, Rockville, MD, USA).

### Flow cytometry

MDA-MB-231 or MDA-MB-468 cells were plated in six-well dishes (1 × 10^6 ^cells per well) either in the presence of anti-Nodal antibody or IgG as described above or left untreated. Antibody or IgG diluted in RPMI was added to the existing volume in each well every 24 hours for a total period of 72 hours. Parallel wells were harvested at 24-hour time-points and subjected to Viacount (cell number) or Annexin V (apoptosis) assays (Guava Technologies/Millipore, Billerica, MA, USA) according to the manufacturer's instructions. Parameters were gated on untreated cells. Within one experiment, each data point was calculated from an average of triplicate samples. Experiments were performed three independent times, and the mean values from three experiments +/- standard error of the mean (SEM) were represented graphically.

### Colony forming assays

Colony forming assays were prepared in triplicate wells with MDA-MB-231 or MDA-MB-468 cells as previously described [17]. Briefly, for each well, 5,000 cells were suspended in 0.35% agarose in complete RPMI or in complete RPMI containing rabbit IgG or anti-Nodal antibody. Suspensions were pipetted onto a solidified layer of 0.5% agarose in complete RPMI in six-well dishes. Cells were cultured for three weeks, then clusters of > 50 cells were scored and photographed using a Zeiss model 25 inverted microscope (Carl Zeiss, Inc.) and Hitachi HV-C20 CCD camera (Hitachi Denshi Ltd., Woodbury, NY, USA).

## Results

### Study group characteristics and Nodal expression in breast tissues

**Figure 1:**
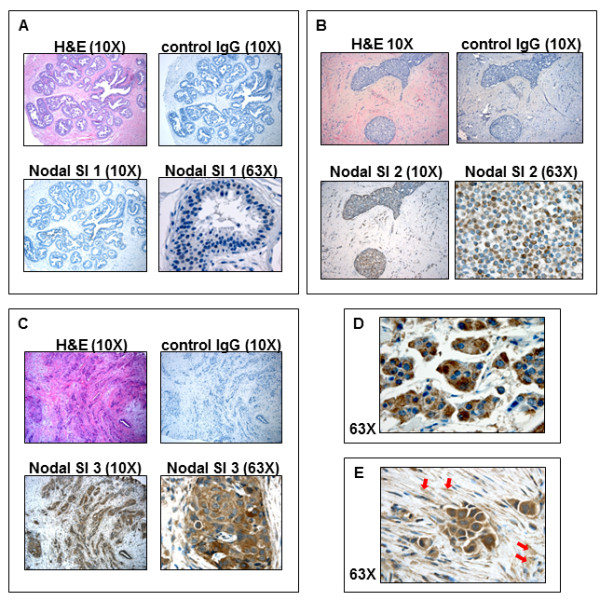
**Immunohistochemistry localization of Nodal in human breast tissues**. Immunohistochemistry staining for Nodal was scored at 10 × and 63 × magnification in samples diagnosed as (**A**) benign breast disease, (**B) **non-invasive, and (**C**) invasive breast cancer. The scores were multiplied to obtain a final Nodal Scoring Index (SI) that ranged from 1 (A) to 9 (C). Nodal staining was generally intracytoplasmic (**D**) and occasionally detected in the surrounding stroma (**E**; red arrows). (original objective magnification indicated in each panel).

Tissue sections from 431 patients determined to have benign or malignant breast disease were studied using immunohistochemistry to evaluate Nodal expression. Patient demographics, including age, race, menopausal and smoking status, are summarized in Table 1. Hematoxylin and eosin (H&E) stained sections were examined at low power magnification and areas of pathologic interest chosen. Nodal Scoring Index (SI) ranged from 0 to 9 as shown in representative images of benign breast disease, non-invasive and invasive breast cancer in Figures 1A-C and grouped into three categories according to the calculated Nodal SI as follows: 0 to 3, 4 to 6 and 9, respectively. Immunohistochemistry staining for Nodal was generally detected in the cytoplasm in a punctate pattern and on the cell membranes of positive cells (Figure 1D). Occasionally, in cases of very strong staining (SI = 9), Nodal was also detected in the surrounding stroma (Figure 1E), suggesting that Nodal may be secreted from Nodal expressing breast cancer cells. Of the 431 total patient samples stained, 143 (33%) showed a Nodal SI of 0 to 3; 213 (49%) had a Nodal SI of 4 to 6; and 75 (17%) a Nodal SI of 9 (Table 1). It is interesting to note that non-smokers tended to have higher Nodal scores (*P *= 0.01).

**Table 1:** Patient characteristics (*N *= 431)

*Characteristic*	*IHC Nodal scoring index category*			*Chi-square* *P-value*
	0 to 3	4 to 6	9	
	N (%)	N (%)	N (%)	
	143 (33)	213 (49)	75 (17)	
*Age group *(*N *= 430)				
< 40	10 (29)	21 (60)	4 (11)	0.741
40-49	32 (35)	43 (47)	16 (18)	
50-59	39 (32)	61 (50)	23 (19)	
60-69	33 (33)	54 (54)	13 (13)	
> = 70	28 (35)	34 (42)	19 (23)	
*Missing	1 (100)	-	-	
*Race*				
White (*N *= 413)	136 (33)	206 (50)	71 (17)	0.96
Other (*N *= 18)	7 (39)	7 (39)	4 (22)	
*Menopausal status*				
Postmenopausal (*N *= 290)	98 (34)	139 (48)	53 (18)	0.64
Other (*N *= 141)	45 (32)	74 (52)	22 (16)	
*Smoking status *(*N *= 426)				
Current	16 (43)	15 (41)	6 (16)	0.011
Former	54 (38)	71 (50)	18 (13)	
Never	69 (28)	126 (51)	51 (21)	
*Missing	4 (80)	1 (20)	-	

*^1 ^*Mantel-Haenszel

* Not included in calculation

### Correlation between Nodal and available clinical data

Table 2 shows a strong association between Nodal SI and biopsy results with malignant breast disease (*N *= 138) showing the greatest percentage for maximum Nodal SI compared to benign breast disease (*N *= 293) (*P *< 0.0001). Nodal expression correlated with the degree of breast cancer differentiation, as assessed by Nottingham Grade (*N *= 104), with higher Nodal SI detected in moderately and poorly differentiated breast cancer tissues compared with well-differentiated breast cancer tissues (*P *= 0.0008) (Table 3). Also, correlation data with tumour stage (*N *= 125) and lymph node stage (*N *= 124) (T-Stage and N-Stage, respectively) showed that patients with advanced (> 1) T-Stage or with lymph node-positive disease had higher Nodal SI (T-Stage *P *= 0.0003 and N-Stage *P *= 0.009, respectively). No significant correlation was observed between Nodal expression and either ER/PR status (*N *= 102) (Table 3) or HER2 expression (*N *= 70) (data not shown).

**Table 2:** Breakdown of Nodal index score by biopsy result (*N *= 431)

*Characteristic*	*IHC Nodal scoring index category*			*Chi-square* *P-value*
	0 to 3	4 to 6	9	
	N (%)	N (%)	N (%)	
*Diagnosis*				
Benign (*N *= 293)	125 (43)	150 (51)	18 (6)	< 0.0001*^1^*
Malignant (*N *= 138)	18 (13)	63 (46)	57 (41)	
*Biopsy result *(*N *= 402)				
Benign	103 (43)	122 (51)	15 (6)	< 0.0001*^1^*
Atypia/Hyperplasia	22 (42)	27 (52)	3 (6)	
Invasive	8 (8)	51 (46)	51 (46)	
*Missing	10 (34)	13 (45)	6 (21)	

*^1 ^*Mantel-Haenszel

* Not included in calculation

**Table 3:** Cancer patient surgery tumour characteristics (*N *= 138)

*Characteristic*	*IHC Nodal Scoring Index Category *			*Chi-Square* *p-value*
	0-3	4-6	9	
	N (%)	N (%)	N (%)	
	18 (13)	63 (46)	57 (41)	
*Pathology *(*N *= 128)				
IDC	7 (7)	41 (45)	44 (48)	0.28*^1^*
DCIS	7 (33)	13 (62)	1 (5)	
Other	0 (0)	7 (47)	8 (53)	
*Missing	4 (40)	2 (20)	4 (40)	
*Nottingham Grade *(*N *= 104)				
Poor	0 (0)	6 (25)	18 (75)	0.0008*^1^*
Moderate	5 (10)	20 (39)	26 (50)	
Well	2 (7)	20 (69)	7 (24)	
*Missing	11 (32)	17 (50)	6 (18)	
*ER/PR Status *(*N *= 102)				
ER or PR Positive	4 (5)	38 (45)	43 (51)	0.43
Negative	3 (18)	6 (35)	8 (47)	
*Missing	11 (31)	19 (53)	6 (17)	
*T-Stage *(*N *= 125)				
DCIS	5 (33)	10 (67)	0 (0)	0.0003*^1^*
Stage 1	7 (9)	39 (48)	36 (44)	
Stage > 1	1 (4)	12 (43)	15 (54)	
*Missing	5 (38)	2 (15)	6 (46)	
*N-Stage *(*N *= 124)				
0	11 (12)	51 (55)	31 (33)	0.009
1	2 (6)	9 (29)	20 (65)	
*Missing	5 (36)	3 (21)	6 (43)	

*^1^*Mantel-Haenszel

* Not included in calculation

### Effects of targeting Nodal in human breast cancer cells *in vitro*

**Figure 2:**
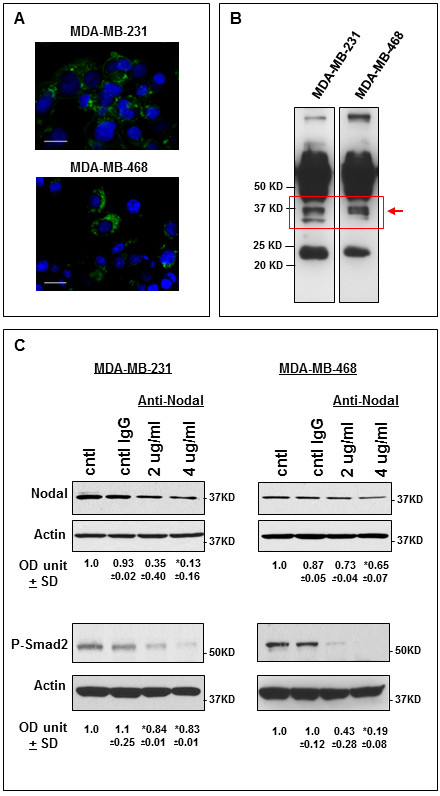
**Nodal expression and effects of Nodal blocking antibody on human breast cancer cells**. In (**A**) immunofluorescence staining confirms Nodal expression in a subpopulation of MDA-MB-231 and MDA-MB-468 human breast cancer cells (bar = 20 μm). Western blotting of conditioned media from MDA-MB-231 and MDA-MB-468 reveals a band in each cell line (red box) corresponding to the molecular weight of pro-Nodal (approximately 36 KD) (**B**). Treatment of human breast cancer cell lines MDA-MB-231 and MDA-MB-468 with Nodal blocking antibody causes a dose dependent reduction in Nodal and P-Smad2 expression, as determined by Western blot analysis, with the most significant reduction observed using 4 μg/ml of Nodal blocking antibody (**C**). (*P < 0.05) (OD unit +/- SD = densitometric units +/- standard deviation).

The expression of Nodal has previously been described in human breast cancer cell lines, including MDA-MB-231 and MDA-MB-468 [17]. The expression of Nodal in these cells was confirmed by immunofluorescence staining and confocal microscopy analysis. Results indicated Nodal expression in 38.6% +/- 2.8% of MDA-MB-231 cells and in 22.2% +/- 5.1% of MDA-MB-468 cells, and shared both cell membrane and intracellular staining patterns (Figure 2A), similar to those described above for the tumour sections (Figure 1D). To determine whether Nodal is secreted by these cells, we evaluated conditioned medium by Western blot analysis for the presence of Nodal protein. We detected bands at approximately 36 KD corresponding to the molecular weight of pro-Nodal [25] in both cell lines, suggesting that Nodal is secreted from breast cancer cells (Figure 2B). This is in agreement with the immunohistochemical detection of Nodal in the extracellular compartment of the patient breast cancer samples (Figure 1E).

To address whether Nodal can be directly targeted in human breast cancer cells, we treated human metastatic MDA-MB-231 and MDA-MB-468 cells with a function blocking anti-Nodal antibody, previously shown to reduce melanoma lung colonization in a Nude mouse model [29]. As Nodal expression is known to be regulated via a positive feedback loop during embryonic development [30], we evaluated the levels of Nodal protein in MDA-MB-231 and MDA-MB-468 cells after 72 hours of treatment with increasing concentrations of anti-Nodal antibody compared with untreated and isotype IgG treated cells. Western blot analyses of whole cell lysates indicate a significant reduction in the abundance of Nodal protein in antibody treated cultures of both cell lines in a dose-dependent manner (Figure 2C). In addition, treatment of both breast cancer cell lines with the Nodal antibody resulted in a significant reduction in phosphorylated Smad-2 levels as determined by Western blot analysis, suggesting a reduction of Nodal downstream signalling in the treated cells compared to non-treated or IgG treated cells (Figure 2C).

**Figure 3:**
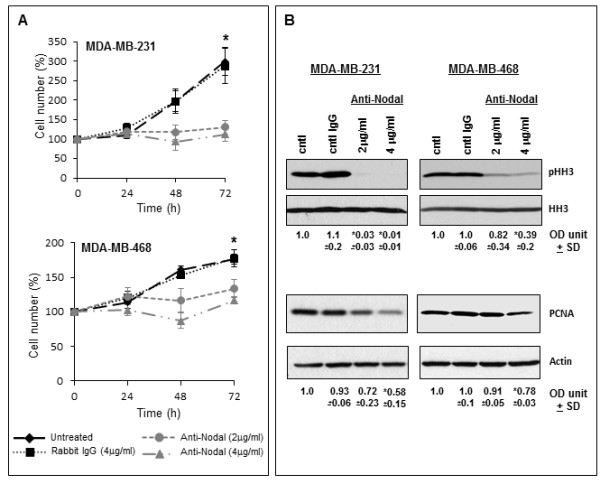
**Effects of Nodal blocking antibody on proliferation of human breast cancer cells**. Nodal blocking antibody caused a dose dependent effect on the proliferation of MDA-MB-231 and MDA-MB-468 breast cancer cells, as determined by flow cytometric analysis (**A**), with the most significant effect observed with 4 μg/ml of the Nodal blocking antibody. The effect on cell proliferation was confirmed by Western blot anlysis of the proliferation markers, phospho-Histone H3 (pHH3) and proliferating cell nuclear antigen (PCNA) (**B**), resulting in the greatest effect with 4 μg/ml of Nodal blocking antibody. (*P < 0.05) (OD unit +/- SD = densitometric units +/- standard deviation).

To determine if Nodal inhibition altered the growth of breast cancer cells, MDA-MB-231 and MDA-MB-468 cell populations were monitored daily by flow cytometry during a period of 72 hours treatment with anti-Nodal antibody. Compared with untreated and IgG treated control cell populations that essentially doubled (MDA-MB-468) or tripled (MDA-MB-231) over the course of the experiment, cell populations treated with anti-Nodal antibody did not increase substantially, and remained significantly diminished compared to control cell populations (Figure 3A). To determine whether this observation was a consequence of a reduction in cell proliferation, the proliferation markers phospho-Histone H3 (pHH3) and proliferating cell nuclear antigen (PCNA) were evaluated by Western blot analysis (Figure 3B). Complementary to the observed reduction in cell growth by flow cytometry, anti-Nodal treated cells exhibited a significant reduction in the cellular levels of Histone H3 phosphorylation, while total Histone H3 (HH3) remained consistent between treatment groups. Furthermore, the cellular expression of PCNA was also significantly reduced in anti-Nodal treated cells from both breast cancer cell lines.

**Figure 4:**
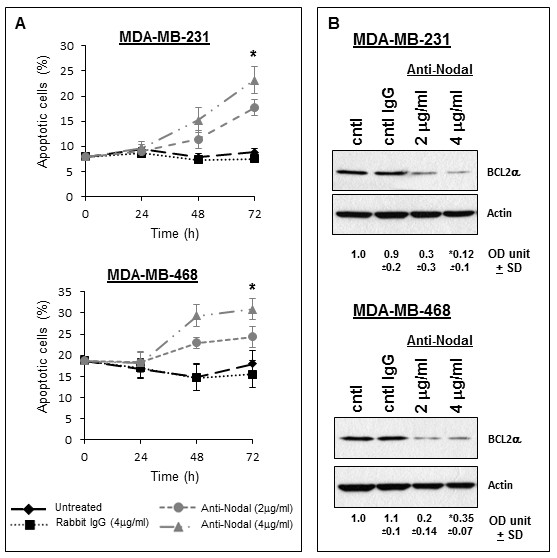
**Nodal blocking antibody reduces human breast cancer cell survival**. A dose dependent increase in apoptosis was observed by flow cytometric analysis with the most significant effect obtained with 4 μg/ml of Nodal blocking antibody in both MDA-MB-231 and MDA-MB-468 human breast cancer cells. This pro-apoptotic effect was confirmed by the reduction in the expression of BCL2α in both MDA-MB-231 and MDA-MB-468 breast cancer cells, which was most pronounced with 4 μg/ml of Nodal blocking antibody. (*P < 0.05) (OD unit +/- SD = densitometric units +/- standard deviation).

**Figure 5:**
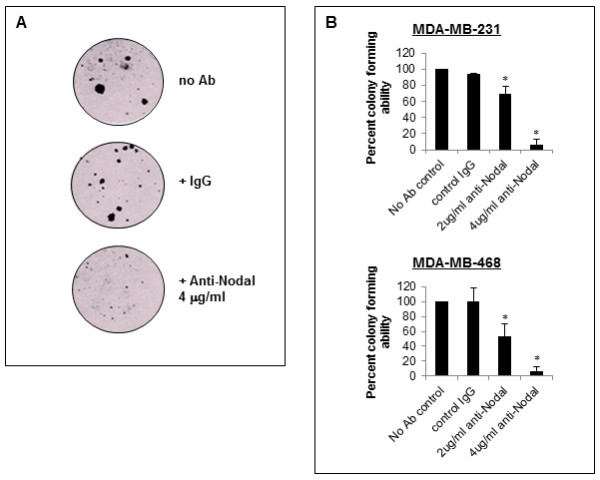
**Nodal blocking antibody affects colony formation of human breast cancer cells**. A representive image of the colony forming assay for MDA-MB-231 is shown in (**A**) (25 × final magnification). A significant reduction in the ability of MDA-MB-231 and MDA-MB-468 cells to form colonies in soft agar was observed using 2 μg/ml and 4 μg/ml of Nodal blocking antibody, compared with no antibody treatment or IgG control (**B**). (*P < 0.05).

To evaluate whether cell death concurrently contributes to the impairment of cell growth observed in anti-Nodal treated cells, apoptosis was measured daily over 72 hours treatment with anti-Nodal antibody using an Annexin V flow cytometry assay (Figure 4A). Compared with untreated and IgG treated control cells that displayed a consistent low level of apoptosis, cells treated with anti-Nodal antibody exhibited a gradual increase in apoptosis over the treatment period that was maximal at 72 hours. While cells treated with the higher dose of anti-Nodal antibody displayed a significant increase in apoptosis at 72 hours compared with untreated and IgG treated control cells, cells treated with the lower dose exhibited an intermediate response. To complement this observation, we also examined the expression of the anti-apoptotic factor BCL2α by Western blotting (Figure 4B). In lysates of cells exposed to the anti-Nodal antibody, the expression of BCL2α was significantly reduced compared with control cells, indicating a shift towards a more pro-apoptotic response in treated cells. Collectively, our data indicate that a consequence of inhibiting Nodal activity in MDA-MB-231 and MDA-MB-468 cells is a combination of impaired cell proliferation and increased apoptosis. Lastly, we measured the effect of Nodal antibody treatment on the ability of human breast cancer cells to form colonies in a 3D soft agar assay (Figure 5A). The results indicate that colony formation by both MDA-MB-231 and MDA-MB-468 cells treated with anti-Nodal blocking antibody was significantly reduced as compared to untreated or IgG treated control cells (Figure 5B), thus demonstrating the efficacy of targeting the tumorigenic potential of Nodal-expressing breast cancer cells.

## Discussion

In this study, immunohistochemistry was used to detect the TGF-beta-related morphogen Nodal in breast tissue samples from over 400 patients (*N *= 431). The analyses revealed the strongest Nodal staining in a significantly greater percentage of patients with malignant breast disease compared to patients with benign breast disease (*P *< 0.0001). Immunofluorescence analysis confirmed Nodal expression in a subpopulation of cells in the two human breast cancer lines studied. Nodal is translated as a precursor form consisting of a signal peptide and pro-domain. Studies using mouse Nodal suggest that the pro-form is subsequently cleaved to a much less stable mature form by extracellular proprotein convertases [31]. Both pro-Nodal and mature Nodal are known to be functionally active [32]. It is impossible to determine which form of Nodal protein is detected in breast tissues and cancer cell lines by immunostaining, since both species are detected by the commercially available Nodal antibody utilized in this study. However, Western blot analysis of cell lysates and conditioned medium from the two breast cancer cell lines detected bands corresponding to the molecular weight of approximately 36 KD reported for pro-Nodal [25]. The smaller mature Nodal (approximately 12-13 KD) was not observed in either cell lysates or conditioned medium, most likely due to the instability of this form. Like most TGF-beta ligands, mature Nodal is capable of forming homodimers; therefore, it is tempting to speculate that the additional band of approximately 24 KD detected in conditioned medium by Western blotting (Figure 2B) could represent a more stable, homodimeric form of mature Nodal. Further studies will be necessary to address whether these secreted form(s) of cancer cell-derived Nodal may affect neighbouring non-Nodal expressing cells. In a previous study, however, we demonstrated that when exogenous recombinant Nodal is added to non-Nodal expressing cells, these cells increase Smad-2 activation [17]. Also, anchorage independent growth that was inhibited in cancer cells treated with Nodal Morpholino treatment, was rescued when these cells were treated with recombinant Nodal protein [16,17], thus supporting the potential for Nodal paracrine effects.

In the category of patients diagnosed with breast cancer (*N *= 138), the degree of Nodal staining showed a significant correlation with poorly differentiated (*P *= 0.008), advanced stage (*P *= 0.0003) and lymph node positive (*P *= 0.009) breast cancer. These observations are in agreement with previous studies describing Nodal expression in aggressive melanoma and glioma and advanced stage endometrial and prostate cancers [16,17,23-25]. Nodal and Cripto-1 (the co-receptor for Nodal) have both been observed in different cancer cell lines, including aggressive human breast cancer cells [17,21]. There is still much debate, however, as to whether Nodal and Cripto-1 can affect cancer cells along distinct pathways or are more likely to function synergistically to propagate downstream signalling events responsible for tumour aggressiveness. Indeed, future studies are needed to specifically address these possibilities. Nevertheless, the present study is the first comprehensive report demonstrating the clinical association between Nodal expression and progression of breast cancer in patient tissues.

Particularly curious is the significant correlation found between higher Nodal expression and non-smoker status (*P *= 0.01). Although it is not clear how smoking can influence the expression of Nodal, it is interesting to note that tobacco smoke has been shown to have deleterious effects on human embryonic stem cells [33]. Thus, one could speculate that since Nodal expression represents the re-emergence of embryonic signalling, initiated perhaps in a subset of breast cancer cells that share certain phenotypic characteristics with stem cells, it is possible that toxicity from tobacco smoke could negatively affect Nodal expression in these stem cell-like breast cancer cells.

It is well established that hormone activity can play a role during tumorigenesis in a variety of responsive tissues [34-37]. Although the relationship between Nodal expression and hormone activity during development, especially in the mammary gland, has not been clearly defined in rigorously controlled studies, a recent report on prostate cancer found that Nodal was capable of reducing the endogenous expression of androgen regulated genes [25]. From this study, one could speculate that a possible effect of Nodal is to regulate differentiation by promoting cell plasticity, which would eventually lead to increased aggressiveness in prostate cancer. In fact, Nodal has been shown to regulate the plastic, endothelial phenotype in melanoma during vasculogenic mimicry [16,28]. Most noteworthy, when Nodal gene expression is down-regulated in tumour cells, the plastic phenotype is diminished, and a more differentiated and less tumorigenic cell phenotype emerges [16,17].

Similar to prostate, hormones play an important role in the development, differentiation and tumorigenesis of breast tissue [38,39]. The expression status of ER or PR in breast cancer represents a useful clinical tool for prognosticating patient survival and predicting the benefit from specific hormonal therapy [40]. However, not all breast cancer patients express these hormone receptors, thus highlighting the need for novel biomarkers that would facilitate universal clinical decisions. Our study did not detect any correlation between Nodal expression and ER or PR status, which was available in 102/138 of the breast cancer cases analysed. However, Nodal was detected in all 138 breast cancer cases, including the samples from patients in which ER or PR status was negative or undetermined. Our results suggest that Nodal could represent a novel biomarker detectable across various stages of breast cancer progression, with the potential to expand the classification scheme based on ER, PR or HER2 status.

Previously, we reported that interference with Nodal signalling can significantly reduce Nodal-dependent cancer cell activities, such as migration and invasion, tumorigenicity and anchorage independent growth [16,17,28,41]. In particular, we showed that it is possible to significantly reduce Nodal expression in human breast cancer cells by exposing them to a human embryonic stem cell conditioned microenvironment containing a Nodal inhibitor, Lefty [17]. Furthermore, knockdown of Nodal with anti-Nodal Morpholino can significantly reduce tumour growth rate and increase apoptosis in an *in vivo *orthotopic human breast cancer xenograft model [41]. Here, we extend these findings by demonstrating that treatment of human metastatic breast cancer cells with a Nodal blocking antibody decreases Nodal expression levels and Smad-2 phosphorylation and reduces cell proliferation and increases apoptosis by reducing cellular levels of pHH3, PCNA and BCL2α. These treatments also led to reduced anchorage independent colony formation in soft agar, further supporting the anti-tumorigenic effect of targeting Nodal. This is in agreement with a previous study where Nodal blocking antibodies were shown to inhibit the colony forming ability of human melanoma cells in soft agar and significantly reduce the ability of these tumour cells to colonize in the lungs of Nude mice [29].

## Conclusions

Our results indicate that the expression of Nodal is associated with advanced stage, invasive human breast cancer. We also describe the inhibitory effects, with underlying mechanistic insights, of a Nodal blocking antibody on human breast cancer cells, extending previous reports showing target validation of Nodal in human cancer. These findings suggest a potential role for Nodal as a novel prognostic biomarker and a promising target for anti-Nodal therapy in breast cancer.

## Abbreviations

CSCs: cancer stem cells; ER: oestrogen receptor; DCIS: ductal carcinoma *in situ*; H&E: hematoxylin and eosin; HER2: human epidermal growth factor receptor 2; HH3: total histone H3; IDC: invasive ductal carcinoma; PCNA: proliferating cell nuclear antigen; pHH3: phospho-histone H3; PR: progesterone receptor; SD: standard deviation; SEM: standard error of the mean; SI: Nodal Scoring Index; STR: short tandem repeat; TGF-beta: tumour growth factor-beta.

## Competing interests

MJCH and EAS hold a patent for Nodal. All other authors have no conflicting interests to declare.

## Authors' contributions

LS participated in the design and coordination of the study, analysis and interpretation of all results, and drafting of the manuscript. KMH participated in the design, execution, acquisition, analysis and interpretation of *in vitro *studies, and helped draft the manuscript. NVM participated in the design, execution, acquisition, analysis and interpretation of all immunohistochemistry results. DWH participated in the design, execution, acquisition, analysis and interpretation of all statistical data. EAS participated in the design, execution, acquisition, analysis and interpretation of *in vitro *studies. BC participated in the pathologic review of patient breast tissue material for immunohistochemical analysis, helped draft the manuscript. XJG: pathologic review of patient breast tissue material for immunohistochemical analysis, and helped draft the manuscript. EAT helped with design and interpretation of *in vitro *results, and helped draft the manuscript. WLL participated in the selection of patient breast tissue material for immunohistochemical analysis, and helped draft the manuscript. CAA participated in the selection of patient breast tissue material for immunohistochemical analysis, and helped draft the manuscript. EAP provided patient cohort; coordinated acquisition and review of clinical data, and helped draft the manuscript. MJCH participated in the conception, design and coordination of the study, and helped draft the manuscript. All authors approved the manuscript.
